# Supplementary material for: A Patient-Derived Scaffold-Based 3D Culture Platform for Head and Neck Cancer: Preserving Tumor Heterogeneity for Personalized Drug Testing
Source: Cells. 2025 Oct 2;14(19):1543. doi: 10.3390/cells14191543 (PMC12524346; doi:10.3390/cells14191543)
Supplement: Supplementary file 1 [file cells-14-01543-s001.zip › supplemental figures_legends.pdf]

Supplementary Figure S2: Characterization of ECM-2 media induced spheroid-like aggregates in 2D cultures. A) Squamous tumor and pEMT cells spontaneously aggregated into nonadherent spheroid-like structures, while CAFs remained attached to the plastic surface. Immunofluorescence staining was performed for epithelial markers (E-cadherin, β-catenin, pan-cytokeratin) and the stromal marker vimentin. B) Same aggregates stained as in A, with alpha smooth muscle actin (α-SMA) replacing Vimentin as a mesenchymal marker, to further validate exclusion of CAFs from the emerging spheroids. Scale bar is 100 μm.

Supplementary Figure S1: **Full, uncropped versions of Western blots for CAF subtype-specific biomarkers.** A) α-smooth muscle actin, B) Vimentin, C) bone-morphometric protein 4 (BMP4), and D) Full-length NOTCH3 protein. Ponceau-S staining is shown below each Western as a loading and transfer control.
