# Supplementary figures and images for: A Patient-Derived Scaffold-Based 3D Culture Platform for Head and Neck Cancer: Preserving Tumor Heterogeneity for Personalized Drug Testing
Source: Cells. 2025 Oct 2;14(19):1543. doi: 10.3390/cells14191543 (PMC12524346; doi:10.3390/cells14191543)

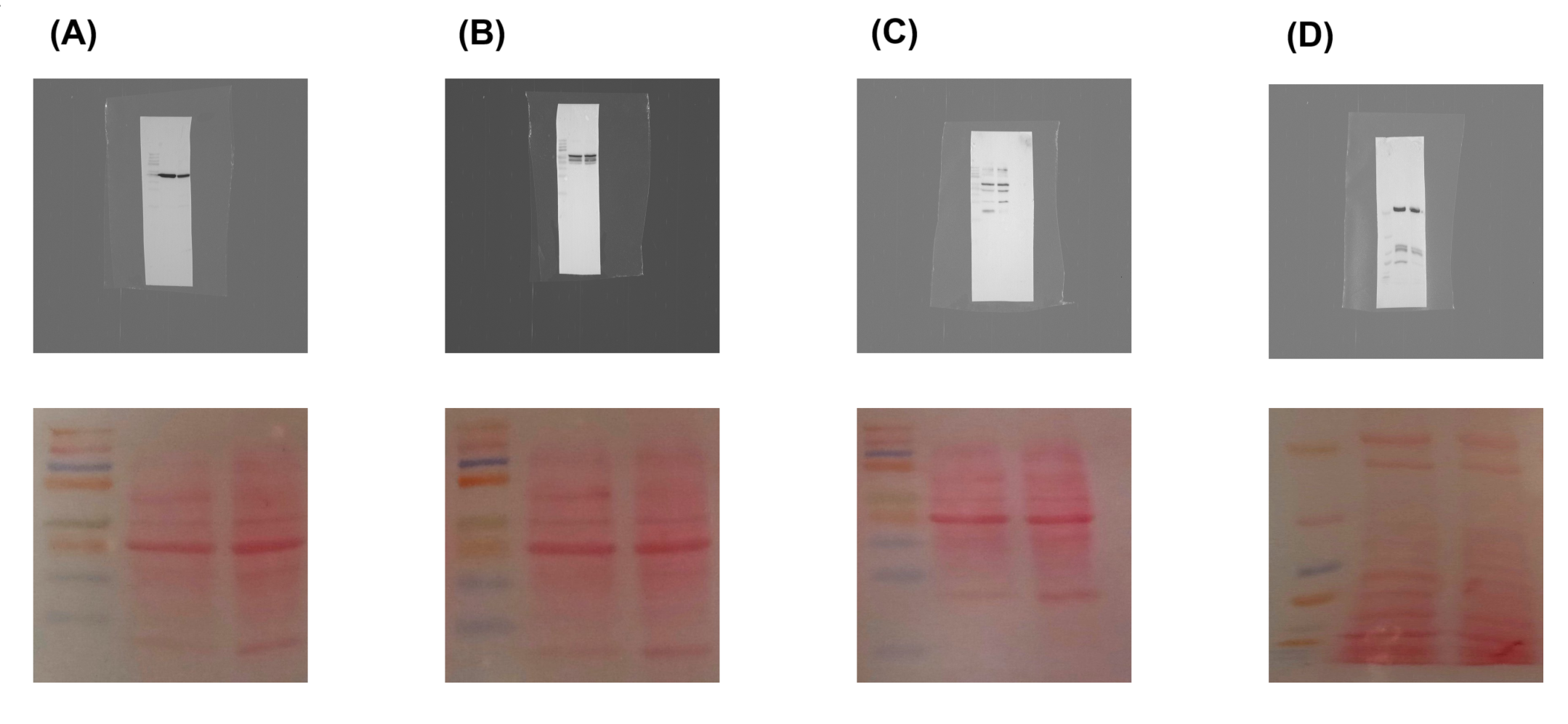

Supplement: Supplementary file 1 [file cells-14-01543-s001.zip › Supplemental Figure 1.png]

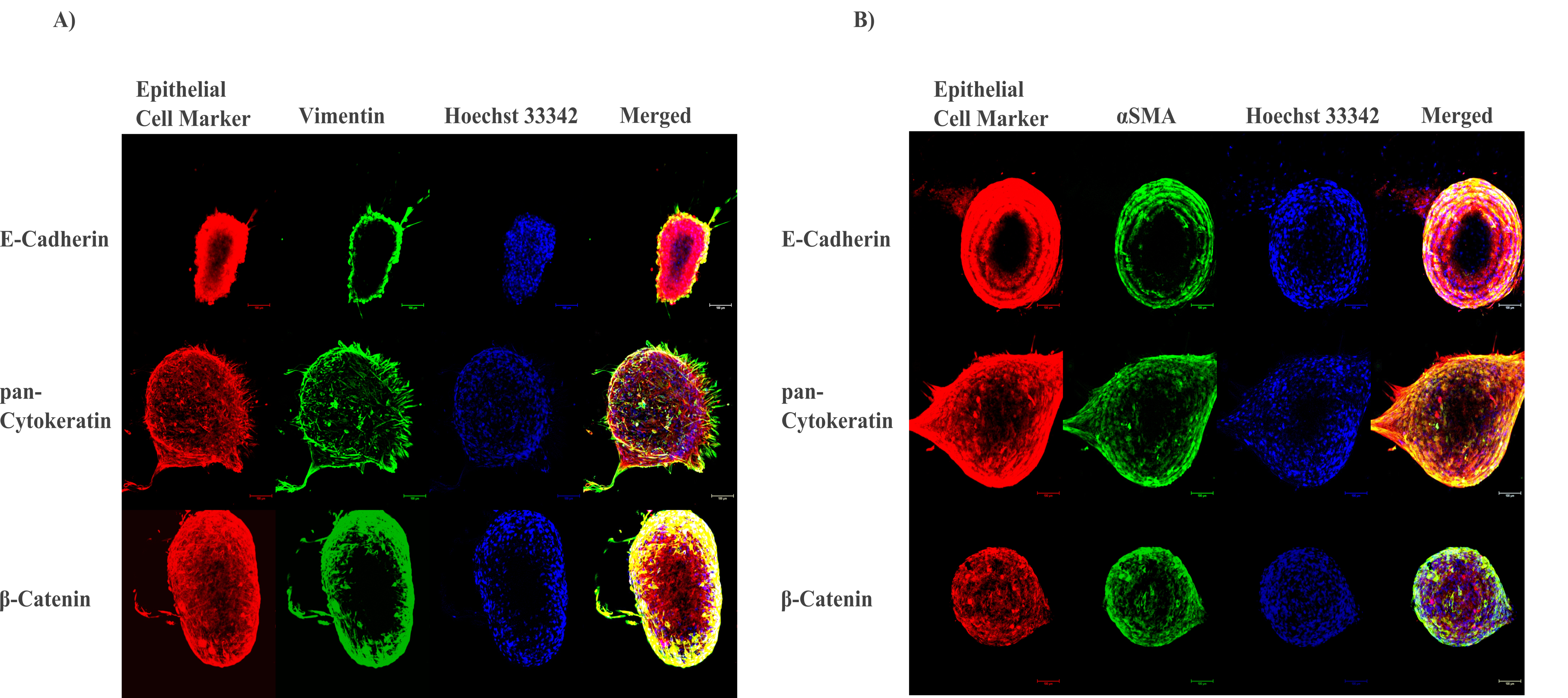

Supplement: Supplementary file 1 [file cells-14-01543-s001.zip › supplementary Figure 2.png]
